# Development and characterization of novel erythropoiesis stimulating protein (NESP)

**DOI:** 10.1054/bjoc.2001.1746

**Published:** 2001-04

**Authors:** J C Egrie, J K Browne

**Affiliations:** Amgen Inc, One Amgen Center Drive, MS 27-4-A, Thousand Oaks, CA 91320-799, USA

**Keywords:** erythropoietin, darbepoetin alfa, pharmacokinetics, biological activity, carbohydrate, review

## Abstract

Studies on human erythropoietin (EPO) demonstrated that there is a direct relationship between the sialic acid-containing carbohydrate content of the molecule and its serum half-life and in vivo biological activity, but an inverse relationship with its receptor-binding affinity. These observations led to the hypothesis that increasing the carbohydrate content, beyond that found naturally, would lead to a molecule with enhanced biological activity. Hyperglycosylated recombinant human EPO (rHuEPO) analogues were developed to test this hypothesis. Darbepoetin alfa (novel erythropoiesis stimulating protein, NESP, ARANESP^TM^, Amgen Inc, Thousand Oaks, CA), which was engineered to contain 5 N-linked carbohydrate chains (two more than rHuEPO), has been evaluated in preclinical animal studies. Due to its increased sialic acid-containing carbohydrate content, NESP is biochemically distinct from rHuEPO, having an increased molecular weight and greater negative charge. Compared with rHuEPO, it has an approximate 3-fold longer serum half-life, greater in vivo potency, and can be administered less frequently to obtain the same biological response. NESP is currently being evaluated in human clinical trials for treatment of anaemia and reduction in its incidence.© 2001 Cance Cance Cancer Research Campaign


*British Journal of Cancer* (2001) **84** (Supplement 1), 3–10
© 2001 Cancer Research Campaign
doi: 10.1054/ bjoc.2001.1746, available online at http://www.idealibrary.com on IDEAL®

### Development and characterization of novel erythropoiesis stimulating protein (NESP)

**JC Egrie and JK Browne**

Amgen Inc, One Amgen Center Drive, MS 27-4-A, Thousand Oaks, CA 91320-1799, USA

**Summary** Studies on human erythropoietin (EPO) demonstrated that there is a direct relationship between the sialic acid-containing carbohydrate content of the molecule and its serum half-life and in vivo biological activity, but an inverse relationship with its receptor-binding affinity. These observations led to the hypothesis that increasing the carbohydrate content, beyond that found naturally, would lead to a molecule with enhanced biological activity. Hyperglycosylated recombinant human EPO (rHuEPO) analogues were developed to test this hypothesis. Darbepoetin alfa (novel erythropoiesis stimulating protein, NESP, ARANESP™, Amgen Inc, Thousand Oaks, CA), which was engineered to contain 5 N-linked carbohydrate chains (two more than rHuEPO), has been evaluated in preclinical animal studies. Due to its increased sialic acid-containing carbohydrate content, NESP is biochemically distinct from rHuEPO, having an increased molecular weight and greater negative charge. Compared with rHuEPO, it has an approximate 3-fold longer serum half-life, greater in vivo potency, and can be administered less frequently to obtain the same biological response. NESP is currently being evaluated in human clinical trials for treatment of anaemia and reduction in its incidence. © 2001 Cancer Research Campaign

**Keywords**: erythropoietin; darbepoetin alfa; pharmacokinetics; biological activity; carbohydrate; review

Erythropoietin (EPO) is a glycoprotein hormone that is the primary regulator of erythropoiesis, maintaining the body's red blood cell mass at an optimum level (Krantz, 1991; Lacombe and Mayeux, 1998). In response to a decrease in tissue oxygenation, EPO synthesis increases in the kidney. The secreted hormone binds to specific receptors on the surface of red blood cell precursors in the bone marrow, leading to their survival, proliferation and differentiation, and ultimately to an increase in haematocrit (D'Andrea et al, 1989; Lodish et al, 1995).

Since its introduction more than a decade ago, recombinant human EPO (rHuEPO) has become the standard of care in treating the anaemia associated with chronic renal failure (CRF). It is highly effective at correcting the anaemia, restoring energy levels, and increasing patient well being and quality of life (Winearls et al, 1986; Eschbach et al, 1987, 1989; Evans et al, 1990). It has also been approved for the treatment of anaemia associated with cancer, HIV infection, and use in the surgical setting to decrease the need for allogeneic blood transfusions. For all indications, it has proven to be remarkably well tolerated and highly efficacious (Markham and Bryson, 1995; Cazzola et al, 1997; Sowade et al, 1998).

The recommended and usual therapy with rHuEPO is 2 to 3 times per week by subcutaneous or intravenous injection. For CRF patients, the duration of therapy is for the life of the patient, or until a successful kidney transplant restores kidney function, including the production of the natural hormone. For cancer patients, rHuEPO therapy is indicated for as long as the anaemia persists, generally through the entire course of chemotherapy.

It can be a hardship to administer rHuEPO 2 to 3 times per week, particularly for those patients who do not otherwise need to be seen in the clinic this frequently. In these cases, the patient needs to make a special trip to the clinic for his or her rHuEPO therapy. As is true for all growth factors, reduction in dose frequency results in a significant loss in efficiency. That is, the total weekly dose required when rHuEPO is administered one time per week is greater than when it is administered as 2 to 3 divided doses. It was anticipated that this clinical need could be addressed by creating a molecule with enhanced in vivo bioactivity to allow for less frequent dosing of patients.

To create a molecule with enhanced activity, research was initially directed towards elucidating those factors and structural features that control the in vivo activity of EPO (Egrie et al, 1993). This research led to the discovery and development of darbepoetin alfa, a novel erythropoiesis stimulating protein (NESP, ARANESP™, Amgen Inc, Thousand Oaks), that can be administered less frequently than epoetin (Egrie et al, 1997).

### STRUCTURE OF EPO

Human EPO is a 30 400 dalton heavily glycosylated protein hormone (Miyake et al, 1977; Davis et al, 1987). Sixty percent (by weight) of the molecule is an invariant 165 amino acid single polypeptide chain containing 2 disulphide bonds (Lai et al, 1986; Recny et al, 1987). The remaining 40% of the mass of the molecule is carbohydrate. Carbohydrate addition (glycosylation) is a post-translational event that results in the addition of sugar chains to specific asparagine (N-linked) or serine/threonine (O-linked) amino acids in the polypeptide. The carbohydrate portion of natural and recombinant human EPO consists of 3 N-linked sugar chains at Asn 24, 38 and 83, and one O-linked (mucin-type) sugar chain at Ser 126 (Browne et al, 1986; Egrie et al, 1986).

*Correspondence to:* JC Egrie

This review article was supported by Amgen Inc, Thousand Oaks, California, USA.

Structure determinations using nuclear magnetic resonance (NMR) spectroscopy (Cheetham et al, 1998) and X-ray crystallography (Syed et al, 1998) have indicated that human EPO is an elongated molecule with an overall topology of a left-handed 4-helix bundle, typical of members of the haematopoietic growth factor family. In addition, these studies have identified the amino acids at the receptor-binding sites. The carbohydrate addition sites are clustered at one end of the molecule, distal from the receptor-binding site. While the 4 carbohydrate chains contribute approximately 40% of the mass of the hormone, they probably cover much of the surface of the molecule since they have an extended and flexible molecular structure.

In contrast to the invariant amino acid sequence of the protein portion of glycoproteins, the carbohydrate structures are variable, a feature referred to as microheterogeneity. For example, N-glycosylation sites on the same protein may contain different carbohydrate structures. Furthermore, even at the same glycosylation site on a given glycoprotein, different structures may be found. This heterogeneity is a consequence of the non-template-directed synthesis of carbohydrates.

The carbohydrate structures of EPO have been determined and the extent of the microheterogeneity defined for both rHuEPO and the natural hormone (Sasaki et al, 1987, 1988; Takeuchi et al, 1988; Tsuda et al, 1988). One of the most prominent examples of microheterogeneity for EPO is seen on the N-linked carbohydrate chains, where the oligosaccharides may contain 2, 3 or 4 branches (or antennae), each of which is typically terminated with the negatively charged sugar molecule, sialic acid (Figure 1). With the exception of sialic acid, all of the other sugar molecules on EPO are neutral. Similarly, the single O-linked carbohydrate may contain 0 to 2 sialic acid molecules. Since each of the 3 N-linked oligosaccharides can contain up to 4 sialic acid residues, and the single O-linked chain can contain 2, the EPO molecule can have a maximum of 14 sialic acid residues. Therefore, because of the variability in sugar structure, the number of sialic acid molecules on EPO varies, and as a consequence, so does the molecule's net negative charge. As indicated in Figure 1, an isoform of EPO is defined as a subset of the EPO molecules that has a defined charge due to its sialic acid content. For reference, epoetin alfa (EPOGEN®, Amgen Inc, Thousand Oaks, CA), the source of the purified rHuEPO used for these studies, has been purified so as to contain isoforms 9–14.

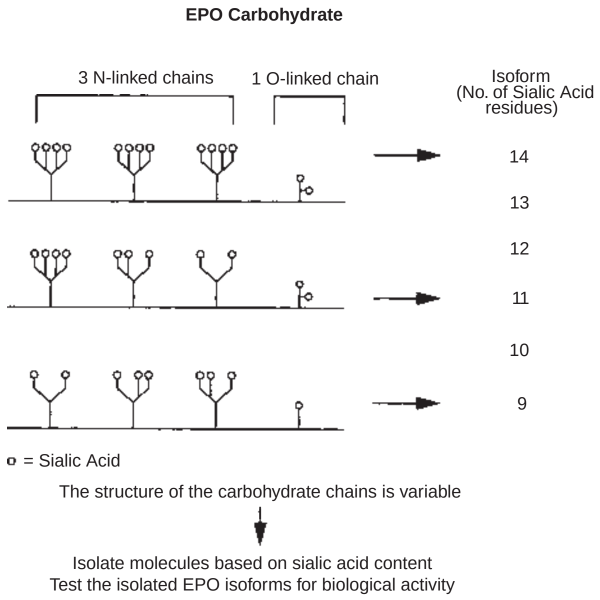

**Figure 1** Schematic of EPO carbohydrate structure and EPO isoform designation

### ROLE OF CARBOHYDRATE IN BIOLOGICAL ACTIVITY

The carbohydrate portions of different glycoprotein molecules have been shown to have many diverse functions, including effects on the biosynthesis and secretion, immune protection, conformation, stability, solubility and biological activity of molecules (Skehel et al, 1984; Cumming, 1991). For rHuEPO, in particular, it has been shown that the addition of carbohydrate is required for secretion from the cell, and for increasing the solubility of the molecule (Dube et al, 1988; Narhi et al, 1991; Delorme et al, 1992). Early research on EPO from natural sources indicated that the sialic acid residues were necessary for biological activity in vivo (Lowry et al, 1960; Lukowsky and Painter, 1972; Goldwasser et al, 1974). Removal of the sialic acid from either native EPO or rHuEPO resulted in molecules having an increased activity in vitro, but very low activity in vivo, presumably due to removal from circulation by the asialoglycoprotein receptor in the liver (Fukuda et al, 1989; Spivak and Hogans, 1989). Similarly, it was shown that EPO molecules, which have been deglycosylated to remove carbohydrate (or produced in *E. coli* to allow expression of only the EPO polypeptide), are active in vitro, but have very low in vivo activity (Dordal et al, 1985; Higuchi et al, 1992).

In order to define further the role of carbohydrate in biological activity, the approach taken was to purify EPO carbohydrate isoforms, measure their in vivo activity and determine how the different carbohydrate structures affect activity.

RHuEPO, produced by Chinese hamster ovary cells, was purified to contain the entire complement of isoforms 4–14, and then further fractionated by ion exchange chromatography to isolate the individual isoforms (Egrie et al, 1993). The in vivo efficacy of each of the individual isoforms was tested in normal mice to determine the effect of repetitive dosing on the haematocrit. In this assay, CD-1 mice were injected with either a vehicle control or an equimolar dose (2.5 mcg $kg^{-1}$ of peptide) of each of the individual isoforms by intraperitoneal injection 3 times per week for 1 month. The results of this experiment demonstrated a striking difference in the biological activity of the individual isoforms, with those isoforms having a higher sialic acid content exhibiting a progressively higher in vivo efficacy (Figure 2). By day 30, the group mean haematocrit of isoform 14-treated animals increased by 26.2 ± 2.7 points (to a haematocrit of 76.2%), compared with an increase of only 6.3 ± 3.5 points for the isoform 8-treated group, a 4.2-fold increase in efficacy. In contrast, animals receiving vehicle control showed no haematocrit change from baseline during the experiment.

It was reasoned that these results might have 2 possible explanations: the more active isoforms might have a longer serum half-life and/or an increased ability to bind to the EPO receptor. In order to assess the contribution of each of these possibilities, the pharmacokinetics and receptor-binding activity of the individual isoforms was measured.

© 2001 Cancer Research Campaign

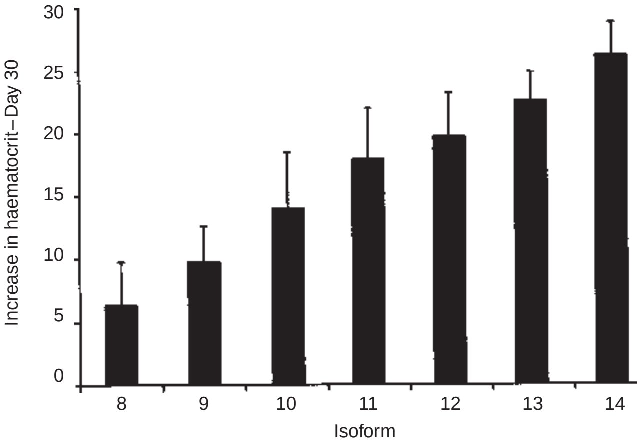

**Figure 2** In vivo efficacy of isolated EPO isoforms. CD-1 mice ($n$ = 20/group) received an equimolar dose (2.5 mcg $kg^{-1}$ of peptide) of each of the isolated EPO isoforms or vehicle control 3 times per week for 30 days by intraperitoneal injection. The haematocrit of each mouse was determined at baseline and twice-weekly thereafter. At the conclusion of the study, final serum samples from all mice were screened for anti-rHuEPO antibodies by a radioimmunoprecipitation assay (Egrie et al, 1986). Mice that developed a significant anti-rHuEPO antibody response were excluded from analysis. The increase in haematocrit over baseline following 30 days of treatment (Day 30) was calculated for each mouse and the group average haematocrit increase (± SD) calculated for each group. There was no significant change in haematocrit in the vehicle control group over the course of the study (46.1% ± 1.2 vs 45.2% ± 1.2 for baseline and day 30, respectively)

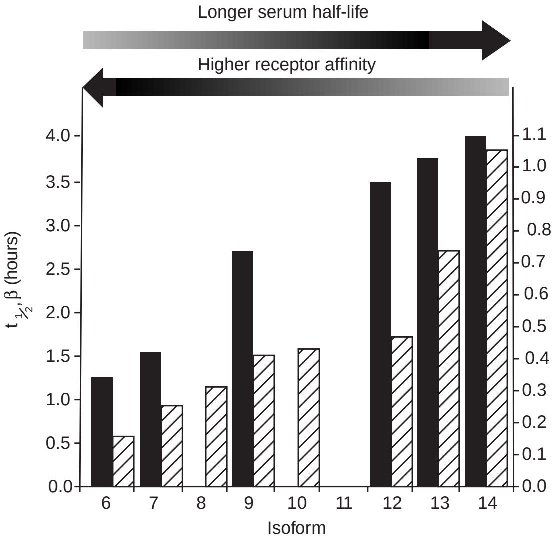

**Figure 3** EPO isoforms having a higher sialic acid content have a longer serum half-life and a lower receptor binding activity. The serum half-life (solid bars) and receptor binding activity (hatched bars) were determined for various isolated EPO isoform preparations. To determine serum half-life, iodinated isoforms were administered intravenously into cannulated Sprague-Dawley rats ($n$ = 4/group). Blood samples were collected and the fraction of ethanol-precipitable iodinated isoform remaining in the serum was measured. The receptor binding activity of isolated EPO isoforms was measured using a radioreceptor assay (Broudy et al, 1988). Increasing concentrations of each unlabelled isolated EPO isoform were incubated with 0.5 ng of $^{125}$I-rHuEPO (a mixture of isoforms 9–14) and OCIM1 cells. Cell receptor-bound $^{125}$I-rHuEPO was measured following separation from unbound $^{125}$I-rHuEPO. Linear regression analysis was used to calculate the $IC_{50}$ (the concentration of unlabelled EPO isoform required to compete 50% of the amount of $^{125}$I-rHuEPO bound in the absence of cold competitor). The $IC_{50}$ for unlabelled rHuEPO was 0.54 ng

The isolated EPO isoforms were iodinated and their circulating half-life determined after intravenous injection into rats. At specified intervals after dosing, blood samples were taken and the fraction of iodinated isoform remaining in circulation was measured. The isoforms with the increased sialic acid content had a longer serum half-life than those with the lower sialic acid content (Figure 3). The beta half-life of isoform 14 was 3.2-fold longer than that for isoform 6 (3.97 vs 1.24 hours, respectively). As expected from these results, the serum clearance of the isoforms progressively increased as the sialic acid content decreased. In contrast, the volume of distribution was the same for the individual isoforms and approximately equal to the plasma volume (data not shown). Thus, those isoforms that have a higher sialic acid content have a higher in vivo biological activity, longer serum half-life and slower serum clearance.

Next, the relative affinity of various EPO isoform preparations for the EPO receptor was determined in a radioreceptor assay (Broudy et al, 1988). This assay measures the quantity of each isoform required to displace $^{125}$I-rHuEPO bound to the EPO receptor on the surface of OCIM1 cells. The $IC_{50}$, the amount of test compound required to compete 50% of the receptor-bound $^{125}$I-rHuEPO, was determined for each isoform. As seen in Figure 3, the higher the sialic acid content, the greater the quantity of EPO isoform necessary to compete $^{125}$l-rHuEPO binding. Thus, those isoforms having a higher sialic acid content had a lower relative affinity for the EPO receptor. The relative affinity of isoform 6 for the EPO receptor was 7-fold greater than that for isoform 14.

Taken together, these experiments indicate that the carbohydrate moieties of EPO have significant effects on the biological activity of the hormone, modulating both receptor affinity and serum clearance. There is a direct relationship between sialic acid content, in vivo biological activity, and serum half-life, but an inverse relationship with receptor affinity. While conventional wisdom might have predicted that increases in receptor affinity would lead to a more active molecule, clearly this is not the case. In fact, as shown in these experiments, isoform 9, which has a 2.6-fold greater affinity for the EPO receptor than isoform 14, has only approximately one-third of the in vivo activity (Figures 2 and 3). These observations clearly demonstrate that clearance has a far stronger influence on in vivo activity than does receptor-binding affinity. Increases in serum half-life were able to overcome the observed decreases in receptor affinity. Thus, for EPO, serum clearance, not receptor-binding affinity, is the primary determinant of in vivo activity.

### DESIGN OF HYPERGLYCOSYLATED RHuEPO ANALOGUES

In addition to identifying serum half-life as a major controlling factor of the in vivo biological activity of EPO, these experiments led to the hypothesis that increasing the sialic acid containing carbohydrate of EPO would increase its serum half-life and thereby the in vivo biological activity of the molecule.

To test this hypothesis, additional N-linked carbohydrate chains were added to the rHuEPO molecule. N-linked carbohydrate is attached to the polypeptide backbone at a consensus sequence for carbohydrate addition (Asn-XXX-Ser/Thr). To introduce new carbohydrate attachment sites into the polypeptide backbone, the DNA sequence of the cloned human EPO gene needed to be modified to code for one or more new consensus sequences. The consensus sequences needed to be added at positions that were compatible with carbohydrate addition. While the consensus

© 2001 Cancer Research Campaign sequence is necessary for carbohydrate addition, it is not sufficient to ensure that a carbohydrate addition site will be utilized. Other factors, such as the local protein folding and conformation during biosynthesis, determine whether an oligosaccharide is attached at a given consensus sequence site. In addition, the consensus sequences needed to be added to positions that did not interfere with receptor binding, or compromise the folding, conformation, or stability of the molecule.

At the time that these studies were initiated, there was only an incomplete understanding of the 3-dimensional structure of EPO. This understanding was largely gained through site-directed mutagenesis studies where individual amino acids were changed and the result of the change evaluated for effects on bioactivity and conformation (Lin, 1987; Boissel and Bunn, 1990) These structure/function experiments identified many of the amino acids that were critical for EPO receptor interaction and necessary for proper folding of the molecule. Although these studies provided insight as to which amino acids should not be altered, they did not allow identification of positions where extra carbohydrate addition sites could be added whilst maintaining structure.

To test this hypothesis, several dozen analogues of rHuEPO containing one or more amino acid substitutions, which created one or more new carbohydrate addition sites, were produced (Elliott et al, 2000). Site-directed mutagenesis was first used to change the nucleic acid sequence encoding one or more amino acids of a human EPO cDNA clone. Next, the clone encoding each new candidate analogue was transfected into mammalian cells and the expressed protein analysed. Only a few of the several dozen analogues tested were fully glycosylated, had the proper tertiary structure, and retained biological activity. The EPO analogues that were properly glycosylated each had one extra N-linked carbohydrate chain (4-chain analogue). By combining the carbohydrate addition sites of 2 successfully glycosylated 4-chain analogues into one molecule, the 5 N-linked chain analogue, NESP, was created. The amino acid sequence of NESP differs from that of human EPO at 5 positions (Ala30Asn, His32Thr, Pro87Val, Trp88Asn, and Pro90Thr) allowing for additional oligosaccharide attachment at asparagine residues at positions 30 and 88 (Elliott et al, 2000).

Due to the additional carbohydrate, these 4 and 5 N-linked (NESP) chain analogues are each biochemically distinct from rHuEPO (Figure 4). They have an increased molecular weight, sialic acid content and negative charge. Each additional N-linked carbohydrate chain increases the molecular weight of the protein by approximately 3300 daltons and adds up to 4 additional sialic acid residues. Thus, in comparison with rHuEPO, the 2 extra carbohydrate chains on NESP increase the molecular weight by 22% (to 37 100 daltons) and the maximum number of sialic acid residues from 14 to 22.

### BIOLOGICAL ACTIVITY OF HYPERGLYCOSYLATED rHuEPO ANALOGUES

These analogues, having four and five N-linked carbohydrate chains, were used to test the hypothesis that increasing the sialic acid-containing carbohydrate content of EPO would increase the serum half-life and thereby the in vivo bioactivity.

The in vivo efficacy of rHuEPO, the 4-chain analogue, and NESP (5-chain analogue) were compared by measuring the increase in haematocrit of mice injected thrice-weekly with equimolar doses (2.5 mcg $kg^{-1}$ of peptide) of each molecule for 6 weeks (Figure 5). Both NESP and the 4-chain analogue produced a faster rate of haematocrit rise and a higher stable plateau haematocrit than rHuEPO. By day 31 the haematocrit had increased by 22.8 ± 2.3, 28.1 ± 6.8, and 33.9 ± 3.4 points for mice treated with rHuEPO, 4-chain analogue, and NESP, respectively and these haematocrit differences were maintained for the duration of the experiment. Consistent with the hypothesis, the magnitude of the haematocrit increase correlated with the number of carbohydrate chains and the sialic acid content of the molecules. Thus, by day 31, treatment with the 5-chain analogue, NESP, increased the haematocrit 11 points more than rHuEPO, while the response produced by the 4-chain analogue was intermediate between that of NESP and rHuEPO.

To confirm that the mechanism of increased activity was due to an increase in the circulating half-life, the pharmacokinetics of rHuEPO, the 4-chain analogue, and NESP were compared. As seen in Figure 6, the greater the sialic acid-containing carbohydrate content of the molecule, the longer its circulating half-life. The beta half-life of both hyperglycosylated molecules was greater than that determined for rHuEPO and for isoform 14, the highest of the naturally-occurring EPO isoforms. In contrast, the volume of distribution was equivalent for the 3 molecules and approximately equal to the plasma volume (data not shown).

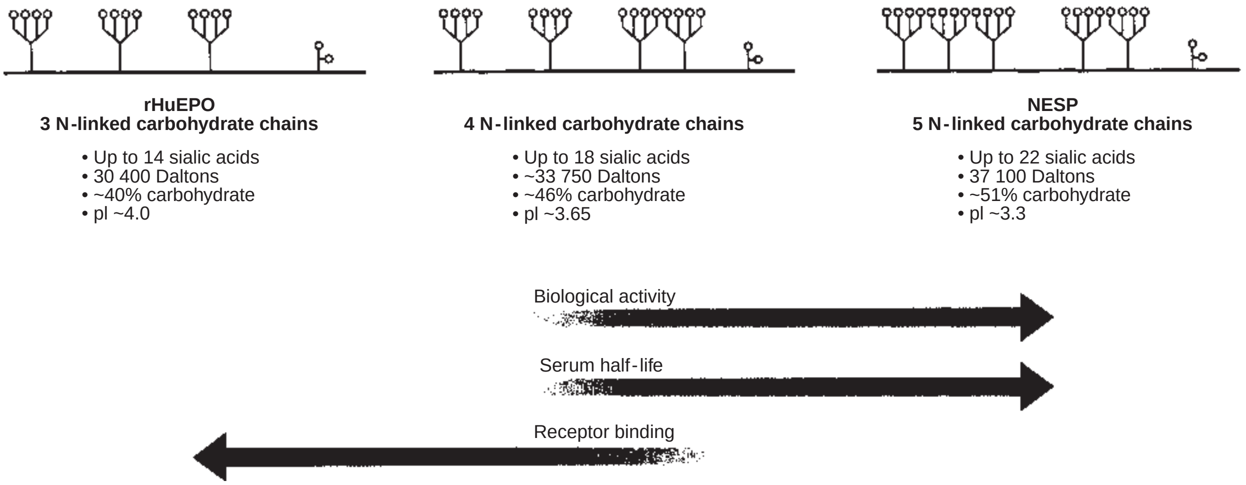

**Figure 4** Biochemical and biological properties of rHuEPO and rHuEPO analogues containing four and five N-linked carbohydrate chains (Egrie et al, 1997)

 © 2001 Cancer Research Campaign

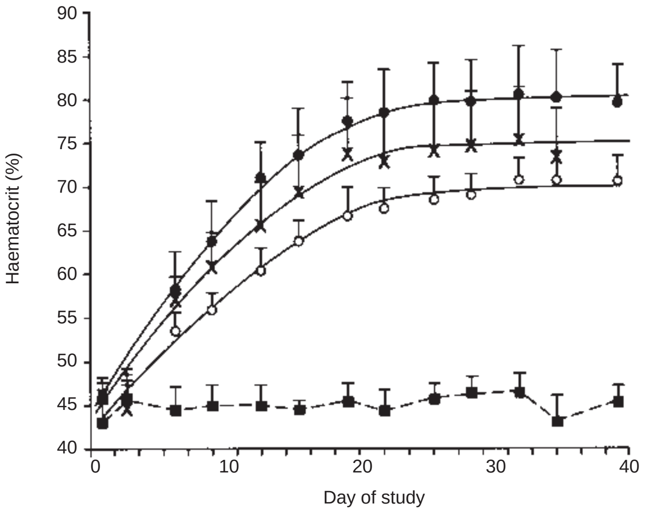

**Figure 5** In vivo efficacy of rHuEPO (isoforms 9–14), 4-chain analogue, and NESP. CD-1 mice (8–13/group) received an equimolar dose (2.5 mcg kg$^{-1}$ of peptide) of rHuEPO (o), 4-chain analogue (x), or NESP (●), or vehicle control (■), 3 times per week for 42 days by intraperitoneal injection. Each point represents the group average haematocrit (± SD) for anti-rHuEPO antibody-negative mice (see Figure 2)

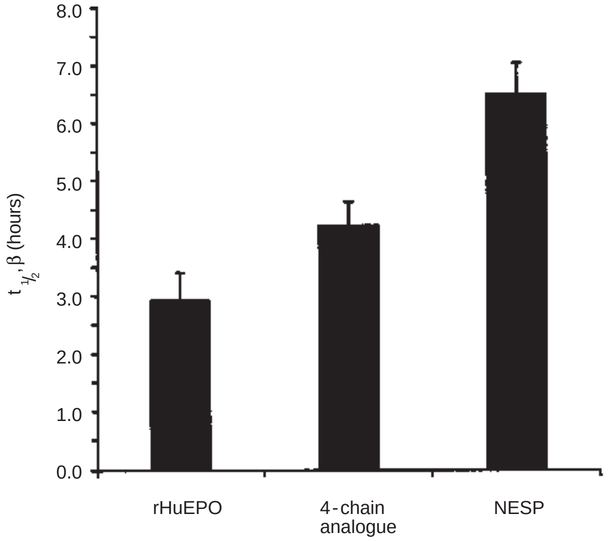

**Figure 6** Serum half-life of rHuEPO (isoforms 9–14), 4-chain analogue, and NESP. The test molecules were iodinated and administered intravenously into cannulated Sprague-Dawley rats (*n* = 10 for rHuEPO, *n* = 6/group for 4-chain analogue and NESP). Blood samples were collected periodically and the amount of ethanol-precipitable iodinated test compound remaining in the serum was measured

RHuEPO, the 4-chain analogue, and NESP were tested for EPO receptor-binding activity in the radioreceptor assay. Consistent with the results obtained in this assay for the isolated isoforms and the hypothesis, the relative EPO receptor-binding affinity was inversely correlated with the carbohydrate content. The relative affinity of NESP for the EPO receptor was 4.3-fold lower than that of rHuEPO and significantly lower than that of isoform 14. The results with the 4-chain analogue were intermediate between those of NESP and rHuEPO (data not shown).

These results confirm the hypothesis that the serum half-life of rHuEPO could be extended by increasing the sialic acid-containing carbohydrate content beyond that found naturally, and that the longer serum half-life would lead to an increase in in vivo biological activity (Figure 4). The results with the 4- and 5-chain hyperglycosylated EPO analogues were also consistent with the previous observations that the increases in serum half-life more than offset the decreases in receptor-binding affinity. The in vivo biological activity of both the 4-chain analogue and NESP were greater than that of rHuEPO even though they each had a lower affinity for the EPO receptor.

### COMPARISON OF THE IN VIVO EFFICACY OF NESP WITH RHuEPO

To further characterize NESP and evaluate its potential as a therapeutic for human use, comparative pharmacodynamic studies of NESP and rHuEPO were performed in normal mice using different frequencies and routes of administration over wide dose ranges. In particular, these studies focused on determining if the increase in serum half-life and in vivo activity would confer the clinical benefit of allowing for less frequent dosing.

As has been shown for rHuEPO, NESP produced a dose-dependent increase in the haematocrit of normal mice when injected by the intravenous, intraperitoneal and subcutaneous routes (Egrie et al, 1997). Initial experiments focused on comparing the efficacy of the 2 molecules when administered 3 times per week. In the experiment shown in Figure 7, thrice-weekly intravenous dosing with 1.25 mcg kg$^{-1}$ of NESP increased the haematocrit of mice to approximately 75% in 6 weeks; however, a comparable haematocrit increase with rHuEPO at this dosing frequency required 5.0 mcg kg$^{-1}$. When the data from all experiments were combined, NESP was determined to be approximately 3.6-fold more potent than rHuEPO when administered three times per week by any route of administration.

NESP can successfully increase the haematocrit of mice when administered once per week (Figure 7). A once-weekly dose of 15 mcg kg$^{-1}$ NESP produced a nearly identical biological response in normal mice as did a thrice-weekly dose of 1.25 mcg kg$^{-1}$ (3.75 mcg kg$^{-1}$ total weekly dose). Thus, for NESP, an approximate 4-fold weekly dose increase is required to change from thrice-weekly to once-weekly dosing in this animal model. It is important to note, however, that in human clinical studies, there were no apparent differences between once-weekly and thrice-weekly dosing with NESP (Macdougall et al, 1998). The optimal weekly dose was the same whether administered once per week or as 3 divided doses. The differences observed between the small animal model, and human clinical trials are presumably due to the differences in the erythrokinetics and red blood cell lifespan of the 2 species.

Once-weekly dosing with rHuEPO is far less efficient than once-weekly dosing with NESP. As shown in Figure 7, once-weekly dosing with 22.5 mcg kg$^{-1}$ rHuEPO increased the haematocrit by 7 points in 6 weeks, while a 2.5 mcg kg$^{-1}$ dose administered three times per week (7.5 mcg kg$^{-1}$ total weekly dose) increased the haematocrit by 22 points in the same time period. Integration of the data from all experiments demonstrated that for rHuEPO an approximate 15-fold weekly dose increase is required to change from thrice-weekly to once-weekly dosing in this animal model.

When the efficacy of once-weekly dosing of NESP and rHuEPO were compared, a striking difference in the potency of the

*© 2001 Cancer Research Campaign* 

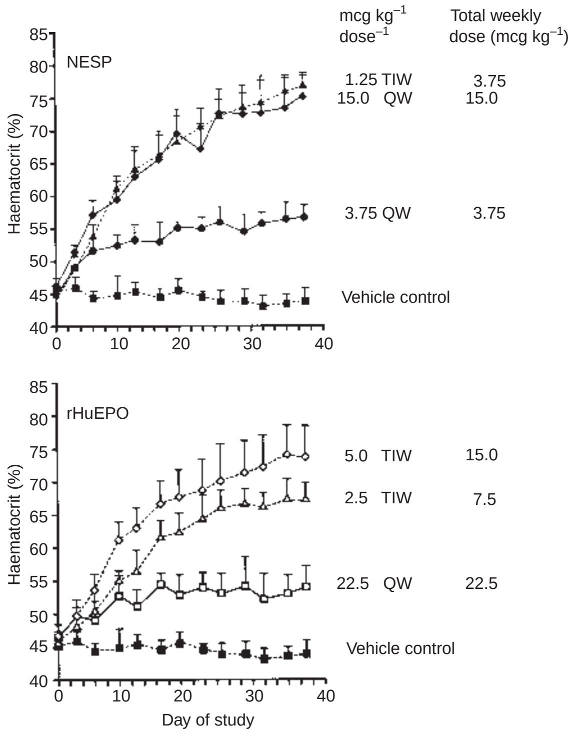

**Figure 7** In vivo efficacy of NESP and rHuEPO administered three times (TIW), or one time (QW) per week. CD-1 mice (*n* = 7/group) received rHuEPO (open symbols), NESP (closed symbols), or vehicle control (■) three times per week (TIW, dashed lines), or one time per week (QW, solid lines) at the indicated dose levels for 42 days by intravenous injection. Each point represents the group average haematocrit (± SD) for the anti-rHuEPO antibody-negative mice (see Figure 2). Both the dose/administration and the total weekly dose are indicated on the graph

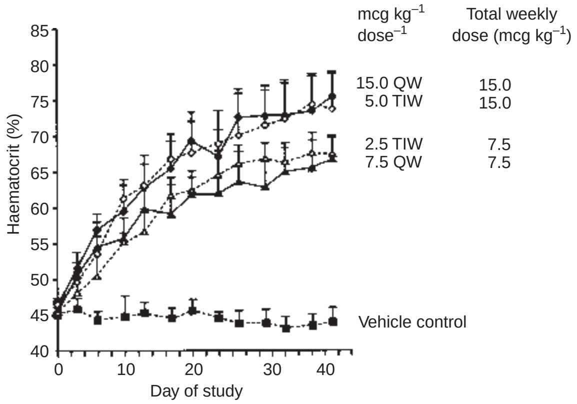

**Figure 8** Comparative efficacy of NESP administered one time per week (QW) with rHuEPO administered three times per week (TIW). CD-1 mice (*n* = 7/group) received rHuEPO (open symbols) three times per week (TIW, dashed lines), NESP (closed symbols) one time per week (QW, solid line), or vehicle control (■) one time per week at the indicated dose levels for 42 days by intravenous injection. Each point represents the group average haematocrit (± SD) for the anti-rHuEPO antibody-negative mice (see Figure 2). Both the dose/administration and the total weekly dose are indicated on the graph two molecules was observed. As seen in Figure 7, a 3.75 mcg kg$^{-1}$ dose of NESP administered one time per week increased the haematocrit by 12 points. In contrast, a 6-fold higher dose of rHuEPO (22.5 mcg kg$^{-1}$) increased the haematocrit by only 7 points. When relative potency plots were constructed from all of the data, NESP was found to be 13- to 14-fold more potent than rHuEPO when each was administered once-weekly.

As a consequence of the relative potency differences described above, NESP administered once-weekly is as effective as the same total weekly dose of rHuEPO given as 3 divided doses (Figure 8). These experiments demonstrate that the same dose of NESP can be administered less frequently than rHuEPO to obtain the same biological response. Furthermore, dosing with NESP as infrequently as once every other week can still increase the haematocrit of normal mice (data not shown).

As expected from the pharmacokinetic differences between NESP and rHuEPO, no one number can be used to express the relative potency difference between the 2 molecules. The relative potency of NESP and rHuEPO will necessarily change as a function of the dosing interval. Longer intervals between the administration of doses will lead to a greater potency difference. Thus, when each molecule is administered 3 times per week NESP is approximately 3.6-fold more potent than rHuEPO; however, when each molecule is administered once-weekly NESP is 13- to 14-fold more potent.

### COMPARATIVE PHARMACOKINETICS OF NESP AND EPOETIN ALFA IN DIALYSIS PATIENTS

The first step in the clinical programme to determine if NESP is both more potent than epoetin alfa and can be administered less frequently, was to verify that NESP had a longer serum half-life in patients. In a double-blind, randomized cross-over study, the single-dose pharmacokinetics of epoetin alfa (100 U kg$^{-1}$) and an equimolar dose of NESP were compared after intravenous administration to 11 stable peritoneal dialysis patients (Macdougall et al, 1999). Serum levels of NESP and rHuEPO were determined at regular intervals up to 96 hours after dosing by immunoassay. In all patients, NESP had a longer terminal half-life than epoetin alfa (Figure 9). The mean terminal half-life for NESP following intravenous injection was 26.3 hours, approximately 3-fold longer than that determined for epoetin alfa (8.5 hours). There was no significant difference in the volume of distribution for the 2 molecules. This first study in man confirmed and extended the observations in animal studies with NESP, demonstrating that due to its increased sialic acid-containing carbohydrate content, NESP has a decreased clearance and longer serum half-life than rHuEPO.

Ongoing clinical studies are evaluating the safety and relative efficacy of NESP as compared with epoetin alfa in different patient groups. Since the amino acid sequence of NESP differs at 5 amino acid positions from EPO, it is theoretically possible that NESP could be immunogenic. If NESP antibodies were to develop, they could be non-neutralizing (benign), or neutralizing, rendering NESP ineffective. In addition, either the neutralizing or non-neutralizing antibodies may cross-react with EPO. Due to this theoretical, but real, concern, patients are being closely monitored for the development of NESP antibodies in all clinical studies.

However, several important features of NESP suggest that the risk of immunogenicity may be minimal. One of the known functions of carbohydrate in glycoproteins is to act as a molecular shield, protecting the underlying polypeptide from the immune

© 2001 Cancer Research Campaign

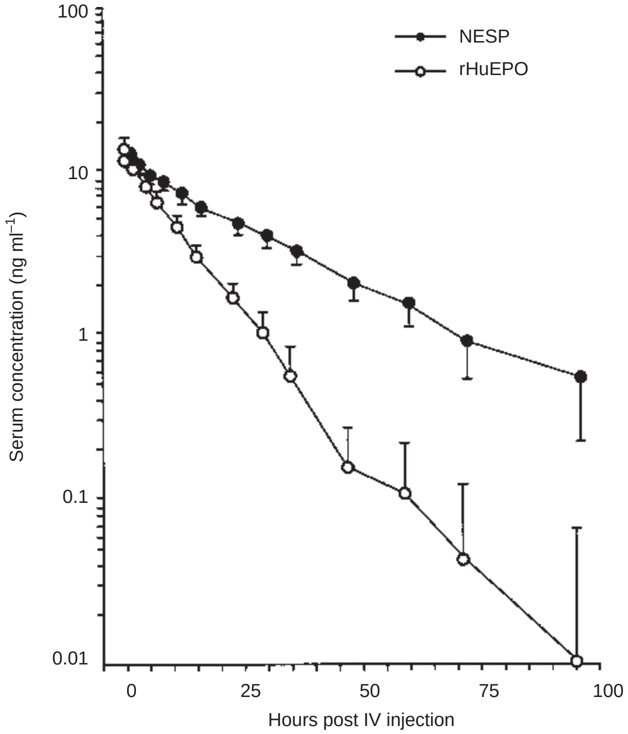

**Figure 9** Comparative pharmacokinetics of NESP and epoetin alfa in anaemic dialysis patients. Results are expressed as the mean (± SD). Reproduced by the kind permission of Lippincott Williams and Wilkins from Macdougall et al, 1999

system (Skehel et al, 1984). Since the location of the 5 amino acid differences between NESP and EPO is at or proximal to the carbohydrate addition site, it is likely that these sites will be well shielded from immune surveillance. The fact that the new carbohydrate addition sites are distal to the receptor-binding site minimizes the possibility that any antibodies that develop would be neutralizing. Carbohydrate chains in themselves are rarely immunogenic and since all of the oligosaccharide structures on NESP are also found on epoetin, it is very unlikely that they will be immunogenic. Results from preclinical studies provide support for these considerations. In a mouse model, where the injection of heterologous human proteins is expected to lead to antibody formation, the incidence of seroconversion in NESP-treated animals was no higher than for rHuEPO-treated animals. Significantly, in all of the clinical trials to date, no patient has developed antibodies to NESP.

### CONCLUSIONS

NESP has been engineered to contain 5 N-linked carbohydrate chains (2 more than rHuEPO). The additional carbohydrate affects the biochemical and biological properties of NESP (Figure 4). Due to the additional sialic acid-containing carbohydrate, NESP has a slower serum clearance and, therefore, a longer half-life than rHuEPO. The longer serum half-life increases the in vivo biological activity and allows NESP to be administered less frequently than rHuEPO. The safety and efficacy of NESP for use as a therapeutic for the treatment of anaemia and reduction in its incidence is being evaluated in ongoing clinical trials.

The development of NESP is an outgrowth of basic research directed towards elucidating those structural features that control the in vivo biological activity of EPO. It was discovered that the pharmacokinetic properties of rHuEPO have a stronger influence on in vivo activity than receptor affinity and that the serum clearance of rHuEPO could be manipulated by changing the proportion of sialic acid-containing carbohydrate. These observations may be applicable for optimization of other protein therapeutics for clinical use.

### REFERENCES

Boissel JP and Bunn HF (1990) Erythropoietin structure-function relationships. In: Dainiak N, Cronkite EP, McCaffrey R, Shadduck RK, eds. *The Biology of Hematopoiesis*. pp 227–232. New York: Wiley-Liss

Broudy VC, Lin N, Egrie J, de Haeen C, Weiss T, Papayannopoulou T and Adamson JW (1988) Identification of the receptor for erythropoietin on human and murine erythroleukemia cells and modulation by phorbol ester and dimethyl sulfoxide. *Proc Natl Acad Sci USA* **85**: 6513–6517

Browne JK, Cohen AM, Egrie JC, Lai PH, Lin FK, Strickland T, Watson E and Stebbing N (1986) Erythropoietin: gene cloning, protein structure, and biological properties. *Cold Spring Harb Symp Quant Biol* **51**: 693–702

Cazzola M, Mercuriali F and Brugnara C (1997) Use of recombinant human erythropoietin outside the setting of uremia. *Blood* **89**: 4248–4267

Cheetham JC, Smith DM, Aoki KH, Stevenson JL, Hoeffel TJ, Syed RS, Egrie J and Harvey TS (1998) NMR structure of human erythropoietin and a comparison with its receptor bound conformation. *Nature Struct Biol* **5**: 861–866

Cumming DA (1991) Glycosylation of recombinant protein therapeutics: control and functional implications. *Glycobiology* **1**: 115–130

D'Andrea AD, Lodish HF and Wong GG (1989) Expression cloning of the murine erythropoietin receptor. *Cell* **57**: 277–285

Davis JM, Arakawa T, Strickland TW and Yphantis DA (1987) Characterization of recombinant human erythropoietin produced in Chinese hamster ovary cells. *Biochemistry* **26**: 2633–2638

Delorme E, Lorenzini T, Giffin J, Martin F, Jacobsen F, Boone T and Elliott S (1992) Role of glycosylation on the secretion and biological activity of erythropoietin. *Biochemistry* **31**: 9871–9876

Dordal MS, Wang FF and Goldwasser E (1985) The role of carbohydrate in erythropoietin action. *Endocrinology* **116**: 2293–2299

Dube S, Fisher JW and Powell JS (1988) Glycosylation at specific sites of erythropoietin is essential for biosynthesis, secretion, and biological function. *J Biol Chem* **263**: 17516–17521

Egrie JC, Strickland TW, Lane J, Aoki K, Cohen AM, Smalling R, Trail G, Lin FK, Browne JK and Hines DK (1986) Characterization and biological effects of recombinant human erythropoietin. *Immunobiology* **172**: 213–224

Egrie JC, Grant JR, Gillies DK, Aoki KH and Strickland TW (1993) The role of carbohydrate on the biological activity of erythropoietin. *Glycoconjugate J* **10**: 263

Egrie JC, Dwyer E, Lykos M, Hitz A and Browne JK (1997) Novel erythropoiesis stimulating protein (NESP) has a longer serum half-life and greater in vivo biological activity than recombinant human erythropoietin (rHuEPO). *Blood* **90**: 56a (abstract 243)

Elliott SG, Lorenzini T, Strickland T, Delorme E and Egrie JC (2000) Rational design of novel erythropoiesis stimulating protein (ARANESP™): a super-sialated molecule with increased biological activity. *Blood* **96**: 82a (abstract 352)

Eschbach JW, Egrie JC, Downing MR, Browne JK and Adamson JW (1987) Correction of the anemia of end-stage renal disease with recombinant human erythropoietin. Results of a combined phase I and II clinical trial. *N Engl J Med* **316**: 73–78

Eschbach JW, Abdulhadi MH, Browne JK, Delano BG, Downing MR, Egrie JC, Evans RW, Friedman EA, Graber SE, Haley NR et al (1989) Recombinant human erythropoietin in anemic patients with end-stage renal disease. Results of a phase III multicenter clinical trial. *Ann Intern Med* **111**: 992–1000

Evans RW, Rader B and Manninen DL (1990) The quality of life of hemodialysis recipients treated with recombinant human erythropoietin. Cooperative Multicenter EPO Clinical Trial Group. *JAMA* **263**: 825–830

Fukuda MN, Sasaki H, Lopez L and Fukuda M (1989) Survival of recombinant erythropoietin in the circulation: the role of carbohydrates. *Blood* **73**: 84–89

Goldwasser E, Kung CK and Eliason J (1974) On the mechanism of erythropoietin-induced differentiation. 13. The role of sialic acid in erythropoietin action. *J Biol Chem* **249**: 4202–4206

Higuchi M, Oh-eda M, Kuboniwa H, Tomonoh K, Shimonaka Y and Ochi N (1992) Role of sugar chains in the expression of the biological activity of human erythropoietin. *J Biol Chem* **267**: 7703–7709
Krantz SB (1991) Erythropoietin. *Blood* **77**: 419–434
Lacombe C and Mayeux P (1998) Biology of erythropoietin. *Haematologica* **83**: 724–732
Lai PH, Everett R, Wang FF, Arakawa T and Goldwasser E (1986) Structural characterization of human erythropoietin. *J Biol Chem* **261**: 3116–3121
Lin FK (1987) The molecular biology of erythropoietin. In: Rich IN, (ed) *Molecular and cellular aspects of erythropoietin and erythropoiesis*. pp. 23–36. New York: Springer
Lodish HF, Hilton DJ, Klingmuller U, Watowich SS and Wu H (1995) The erythropoietin receptor: biogenesis, dimerization, and intracellular signal transduction. *Cold Spring Harb Symp Quant Biol* **60**: 93–104
Lowry PH, Keighley G and Borsook H (1960) Inactivation of erythropoietin by neuraminidase and mild substitution reactions. *Nature* **185**: 102–103
Lukowsky WA and Painter RH (1972) Studies on the role of sialic acid in the physical and biological properties of erythropoietin. *Can J Biochem* **50**: 909–917
Macdougall IC, on behalf of the ARANESP™ UK Study Group (1998) Novel erythropoiesis stimulating protein (NESP) for the treatment of renal anaemia. *J Am Soc Nephrol* **9**: 258a–259a (abstract A1317)
Macdougall IC, Gray SJ, Elston O, Breen C, Jenkins B, Browne J and Egrie J (1999) Pharmacokinetics of novel erythropoiesis stimulating protein compared with epoetin alfa in dialysis patients. *J Am Soc Nephrol* **10**: 2392–2395
Markham A and Bryson HM (1995) Epoetin alfa. A review of its pharmacodynamic and pharmacokinetic properties and therapeutic use in nonrenal applications. *Drugs* **49**: 232–254
Miyake T, Kung CK and Goldwasser E (1977) Purification of human erythropoietin. *J Biol Chem* **252**: 5558–5564
Narhi LO, Arakawa T, Aoki KH, Elmore R, Rohde MF, Boone T and Strickland TW (1991) The effect of carbohydrate on the structure and stability of erythropoietin. *J Biol Chem* **266**: 23022–23026
Recny MA, Scoble HA and Kim Y (1987) Structural characterization of natural human urinary and recombinant DNA-derived erythropoietin. Identification of des-arginine 166 erythropoietin. *J Biol Chem* **262**: 17156–17163
Sasaki H, Bothner B, Dell A and Fukuda M (1987) Carbohydrate structure of erythropoietin expressed in Chinese hamster ovary cells by a human erythropoietin cDNA. *J Biol Chem* **262**: 12059–12076
Sasaki H, Ochi N, Dell A and Fukuda M (1988) Site-specific glycosylation of human recombinant erythropoietin: analysis of glycopeptides or peptides at each glycosylation site by fast atom bombardment mass spectrometry. *Biochemistry* **27**: 8618–8626
Skehel JJ, Stevens DJ, Daniels RS, Douglas AR, Knossow M, Wilson IA and Wiley DC (1984) A carbohydrate side chain on hemagglutinins of Hong Kong influenza viruses inhibits recognition by a monoclonal antibody. *Proc Natl Acad Sci USA* **81**: 1779–1783
Sowade B, Sowade O, Moecks J, Franke W and Warnke H (1998) The safety of treatment with recombinant human erythropoietin in clinical use: a review of controlled studies. *Int J Mol Med* **1**: 303–314
Spivak JL and Hogans BB (1989) The in vivo metabolism of recombinant human erythropoietin in the rat. *Blood* **73**: 90–99
Syed RS, Reid SW, Li C, Cheetham JC, Aoki KH, Liu B, Zhan H, Osslund TD, Chirino AJ, Zhang J, Finer-Moore J, Elliott S, Sitney K, Katz BA, Matthews DJ, Wendoloski JJ, Egrie J and Stroud RM (1998) Efficiency of signalling through cytokine receptors depends critically on receptor orientation. *Nature* **395**: 511–516
Takeuchi M, Takasaki S, Miyazaki H, Kato T, Hoshi S, Kochibe N and Kobata A (1988) Comparative study of the asparagine-linked sugar chains of human erythropoietins purified from urine and the culture medium of recombinant Chinese hamster ovary cells. *J Biol Chem* **263**: 3657–3663
Tsuda E, Goto M, Murakami A, Akai K, Ueda M, Kawanishi G, Takahashi N, Sasaki R, Chiba H, Ishihara H et al (1988) Comparative structural study of N-linked oligosaccharides of urinary and recombinant erythropoietins. *Biochemistry* **27**: 5646–5654
Winearls CG, Oliver DO, Pippard MJ, Reid C, Downing MR and Cotes PM (1986) Effect of human erythropoietin derived from recombinant DNA on the anaemia of patients maintained by chronic haemodialysis. *Lancet* **2**: 1175–1178

 *© 2001 Cancer Research Campaign*
